# Integrin Inhibitors as a Therapeutic Agent for Ovarian Cancer

**DOI:** 10.1155/2012/915140

**Published:** 2011-12-25

**Authors:** Kenjiro Sawada, Chifumi Ohyagi-Hara, Tadashi Kimura, Ken-ichirou Morishige

**Affiliations:** ^1^Department of Obstetrics and Gynecology, Osaka University Graduate School of Medicine, Osaka 565-0871, Japan; ^2^Department of Obstetrics and Gynecology, Gifu University Graduate School of Medicine, 1-1 Yanagido, Gifu 501-1194, Japan

## Abstract

Ovarian cancer is a deadly disease, with a cure rate of only 30%. Despite aggressive treatments, relapse remains almost inevitable in patients with advanced-stage disease. In recent years, great progress has been made towards targeting integrins in cancer treatment, and clinical studies with various integrin inhibitors have demonstrated their effectiveness in blocking cancer progression. Given that the initial critical step of ovarian cancer metastasis is the attachment of cancer cells onto the peritoneum or omentum, in addition to the proven positive clinical results of anti-angiogenic therapy, targeting integrins is likely to be one of the most feasible approaches. This paper summarizes the current understanding of the integrin biology in ovarian cancer metastasis and the various therapeutic approaches attempted with integrin inhibitors. Although no integrin inhibitors have shown favorable results so far, integrin-targeted therapies continue to be a promising approach to be explored for further clinical investigation.

## 1. Introduction

Ovarian cancer is a highly metastatic disease characterized by widespread peritoneal dissemination and ascites and is the leading cause of death from gynecologic malignancies. It is often diagnosed at a late stage after tumor cells are disseminated within the peritoneal cavity. Despite aggressive treatments which consist of surgical cytoreduction and chemotherapy, more than two-thirds of all patients succumb to the disease within 5 years [1]. The initial step of ovarian cancer metastasis is that cancer cells, detached from the ovarian surface epithelium, attach to the layer of mesothelial cells that line the inner surface of the peritoneum. Several integrins have been identified as important mediators of ovarian carcinoma metastasis to the mesothelium, suggesting that integrin inhibitors could be a new therapeutic strategy to prevent cancer cells from attaching onto the peritoneal cavity. During the last 10 years, novel insights into the mechanisms that regulate cell survival as well as cell migration and invasion have led to the development of novel integrin inhibitors for cancer treatments [2]. In this short review, we describe the critical roles of integrins during the metastatic process of ovarian carcinoma and discuss the potential of integrin inhibitors as a new therapeutic agent for the treatment of ovarian cancer.

## 2. Biology of Integrin

The role of integrins in cell migration and invasion is one of their most studied functions in tumor biology [3, 4]. Integrins are cellular surface glycoprotein receptors consisting of a heterodimer of *α*- and *β*-subunits that are mutually non-covalently associated. In mammals, integrins have extensive distributions throughout the whole body, and there are 18 *α*- and 8 *β*-subunits assembling 24 functionally different heterodimers [5, 6]. Each individual integrin subunit has a large extracellular domain, a single membrane-spanning domain and a short noncatalytic cytoplasmic tail. The assembled integrin heterodimer can bind to a unique set of ligands. Natural integrin ligands include the components of the extracellular matrix (ECM) such as collagen, laminin, fibronectin, and vitronectin. Many integrins bind their ligands by recognizing the short amino acid sequences on exposed loops, such as Arg-Gly-Asp (RGD) (integrin *α*5*β*1) or Arg-Glu-Asp-Val (REDV) (integrin *α*4*β*1). On ligation to the ECM, integrins recruit complex signaling events, alone or in combination with growth factor receptors. Integrin signaling regulates diverse functions in tumor cells, including migration, invasion, proliferation, and survival through the activation of various pathways, such as integrin-linked kinase (ILK), mitogen-activated protein kinase (MAPK), protein kinase B (PKB/Akt), or nuclear factor kappa B (NF-*κ*B) [7]. In recent years, great progress has been made towards targeting integrins in cancer treatment. Preclinical as well as clinical studies with various integrin antagonists have demonstrated their effectiveness in blocking tumor progression [3]. Almost all such Phase 1 clinical trials showed that the integrin inhibitors are nontoxic and well tolerated by patients, suggesting that they can be used concurrently with the conventional cytotoxic chemotherapy or radiotherapy. Some reports showed that radiotherapy results in up-regulation of integrin expression in several types of cancer, leading to cellular resistance to radiotherapy-induced cancer cell death [8, 9]. Nam et al. demonstrated in their preclinical works that targeting *β*1-integrin enhances the efficacy of radiation therapy in several cancers including breast cancer [9]. Integrins are also involved in innate multidrug resistance, allowing tumor cells to survive chemotherapy (cell-adhesion-mediated drug resistance: CAM-DR) [8]. It has been proposed that CAM-DR is caused by the activation of *β*1-integrin-stimulated tyrosine kinase that suppresses apoptosis from chemotherapy [10, 11]. Integrin-targeted therapies in addition to conventional cytotoxic treatments, thus, have great potential to enhance the efficacy of overall treatments with minimal side effects. 

## 3. Ovarian Cancer Metastasis and Current Treatment Options

In 2010, the American Cancer Society estimated that there were 21,880 cases of epithelial ovarian carcinoma and 13,850 disease-related deaths, identifying that ovarian cancer has the highest mortality rate of all gynecologic tumors. Sixty-three percent of all patients with ovarian carcinoma will succumb to their disease, making it the fifth leading cause of cancer death among USA women [12]. The high mortality of this tumor is largely explained by the fact that the majority of patients present at an advanced stage, with widespread metastatic disease within the peritoneal cavity. Only 20% of ovarian cancers are diagnosed while they are still limited to the ovaries, and patients at this early stage have an 85–90 percent 5-year survival [13].

In spite of several efforts made for early screening of ovarian cancer, no effective screening methods have been established to reduce ovarian cancer incidence and mortality [14]. Current treatment strategies for advanced ovarian carcinoma consist of aggressive “cytoreductive” or “tumor-debulking” surgery, followed by a combination of platinum- and taxane-based chemotherapy. The surgical treatment goal is “optimal” surgical cytoreduction, which is generally defined as residual disease of 1 cm or less. No gross residual tumors should be left throughout the abdominal cavity, because several studies have convincingly shown that cytoreduction results in improved patient survival [15, 16]. This effect of cytoreduction is indicative of a dramatic difference in the biological behavior of ovarian cancer as compared with other malignancies, because in most other cancers the removal of metastatic tumors does not necessarily lead to improved survival [13]. One of the main reasons for this difference is that, unlike other malignancies, ovarian cancer directly disseminates within the abdominal cavity and rarely disseminates through the vasculature unlike other malignancies, although metastasis in pelvic and/or para-aortic lymph nodes can be found occasionally [17]. Once the cancer cells have detached as single cells or clusters from the primary ovarian tumor, it is thought that they metastasize through a passive mechanism, carried by the physiological movement of peritoneal fluid to the peritoneum and omentum. However, in spite of the execution of primary aggressive cytoreductive surgery as well as meticulously-designed chemotherapy regimens, the overall cure rate of ovarian cancer patients remains approximately 30% [13]. Even though no apparent tumors remain throughout the peritoneal cavity after the initial surgery, invisible cancer cells are left and endure through the postoperative chemotherapy. Small numbers of drug-resistant cells can persist for many months and remain dormant in the peritoneal cavity, only to grow progressively, leading to death of the patient despite aggressive treatment of the recurrent disease. There is, thus, a critical need for novel targeted therapies to overcome this situation. In particular, efficacious consolidation or maintenance therapy after the cytoreduction surgery needs to be explored.

Novel molecularly directed therapies which aim to target tumor cells and the tumor microenvironment in ovarian tumorigenesis are rapidly emerging. Antiangiogenic agents have led the field so far. Preclinical and clinical studies have demonstrated the efficacy of antiangiogenic approaches against ovarian cancer both alone and in combination with cytotoxic chemotherapy [18]. Bevacizumab, a humanized monoclonal antibody directed against VEGF, has been tested in several epithelial malignancies, including ovarian cancer. Several prospective Phase II trials have shown that bevacizumab in combination with chemotherapy (carboplatin-paclitaxel, cyclophosphamide, or topotecan) is efficacious in advanced ovarian cancer [19], and Phase III evaluation is currently ongoing. Although these results are promising and it appears to be clear that bevacizumab is efficacious in a subset of ovarian cancer patients, resistance to bevacizumab is a major obstacle even for patients in whom bevacizumab was initially efficacious [18]. One potential alternative treatment option is targeting integrins, which regulate diverse functions in tumor cells including adhesion, migration, invasion, proliferation, and survival. In addition to tumor cells, integrins are also found on tumor-associated host cells, such as the vascular endothelium, fibroblasts, or bone marrow-derived cells. Targeting integrin signaling has the potential to inhibit the contribution of these cell types to cancer progression [3]. Several integrin-targeted therapeutic agents are emerging and currently in clinical trials for cancer therapy including ovarian carcinoma.

## 4. Integrin Biology in Ovarian Cancer

Most ovarian cancer cells are derived from the epithelial cells that cover the surface of the ovary [1]. Before the ovarian carcinoma cells detach from the basement membrane, they often undergo an epithelial-mesenchymal transition (EMT), which loosens the intercellular adhesions between the cancer cells. EMT often starts from the loss of E-cadherin, one of the molecules crucial for the adhesion between neighboring epithelial cells. During the process of EMT, cancer cells acquire a more invasive phenotype and proliferate and spread throughout the abdominal cavity, carried by the physiological movement of massive ascites. Indeed, the knockdown of E-cadherin was reported to induce the up-regulation of the fibronectin receptor, *α*5*β*1-integrin, which promotes the adhesion of ovarian cancer cells to secondary metastasis sites, such as omentum and peritoneum [17, 20]. According to an immunohistochemical analysis using clinical samples conducted at the University of Chicago (Chicago, IL), about 40% (42 of 107) advanced (Stages II–IV) ovarian cancer patients showed *α*5*β*1-integrin positive staining. Among these positive cases, 10 cases (9%) were considered to show overexpression and the median survival of the patients with *α*5*β*1-integrin overexpression was significantly worse (26 months) than that of those with low or negative integrin expression (35 months) [20]. Once the cancer cells have detached from the primary tumor, they float in the ascites as single cells or as multicellular spheroids. Casey et al. reported that the *β*1-integrin stimulating antibody or exogenous treatment with fibronectin promoted the spheroid formation of ovarian cancer cells, while blocking antibodies against *α*5- or *β*1-integrin inhibited the formation, indicating that interactions between *α*5*β*1-integrin and fibronectin mediate the formation of ovarian carcinoma spheroids and their adhesion to ECMs at the secondary tumor growth sites [21]. The initial key step of ovarian cancer metastasis is the attachment of ovarian cancer cells onto the layer of mesothelial cells which cover the peritoneal cavity. Integrins have also been identified as important mediators between ovarian carcinoma and the mesothelium. Strobel and Cannistra reported that blocking antibodies against *α*5- and *β*1-Integrin as well as RGD peptide inhibited the binding of ovarian cancer cells to mesothelial cells, suggesting that *α*5*β*1-integrin was the major receptor responsible for fibronectin-mediated ovarian cancer binding to the mesothelium [22]. These accumulating results strongly suggest that inhibition of *α*5*β*1-integrin is a potential new therapeutic target, at least for a subset of ovarian cancer patients [21]. Not only fibronectin but also collagen and laminin are the most abundant extracellular proteins in the mesothelium covering the peritoneum and the omentum. Primary ovarian carcinoma cells adhere preferentially to type I collagen, which can be blocked with an *α*2*β*1-integrin antibody [23]. The other important adhesion molecules which interact with cancer cells and the mesothelial cells are *α*4*β*1-integrin and its adhesion receptor, cell adhesion molecule-1 (VCAM-1) [24]. *α*4*β*1-integrin expressed on ovarian carcinoma cells binds to VCAM-1, which is present on the mesothelial cells and function-blocking antibodies directed against VCAM-1 and *α*4*β*1-integrin block migration and metastasis in a xenograft model [24]. The expression of *α*v*β*6 integrin in ovarian cancer cell lines correlates with the invasive potential of cells by inducing the secretion of proteinases such as urokinase plasmin activator (uPA) and matrix metalloproteinases (MMPs) [25]. Inconsistent results have been reported regarding the role of the vitronectin receptor, *α*v*β*3-integrin, in ovarian cancer metastasis. Although it was initially thought to be expressed on aggressive ovarian cancer cells and to be correlated with ovarian cancer cell adhesive, migratory, and proliferative properties, recent data question this assertion and indicate that it is expressed on well-differentiated tumors and acts as a tumor suppressor in ovarian cancer [17]. Kaur et al. reported that *α*v*β*3-integrin-expressing ovarian cancer cells showed impaired invasion, protease expression, and colony formation and that patients with tumors expressing high levels of *β*3-integrin had significantly better prognoses [26]. Given that Reynolds et al. recently showed that nanomolar concentrations of RGD-mimetic *α*v*β*3-/*α*v*β*5-integrin inhibitors enhance tumor growth and tumor angiogenesis in preclinical xenograft models [27], therapies aimed at blocking *α*v*β*3-integrin may have detrimental effects. 

## 5. Clinical Trials Targeting Ovarian Cancer

Preclinical studies have shown that integrin antagonists inhibit tumor growth by affecting both tumor cells and tumor-associated host cells, especially the angiogenic endothelium. Integrin antagonists currently in clinical trials include monoclonal antibodies and Arg-Gly-Asp (RGD) peptide mimetics [3, 31]. The candidate integrin inhibitors which could be applied for ovarian cancer treatment are summarized in Table 1 [20, 26, 29, 30]. Volociximab, a chimeric monoclonal antibody directed against *α*5*β*1-integrin, inhibits angiogenesis and impedes tumor growth. Bell-McGuinn et al. reported on their Phase II data of platinum-resistant ovarian cancer patients treated with volociximab as a monotherapy [32]. Of 14 patients who were evaluable for efficacy, only one patient had stable disease at 8 weeks, and the remaining 13 progressed on treatment, although weekly volociximab was well tolerated. Beside the antibodies, synthetic peptides that mimic the structure of natural integrin binding ligands are alternative candidates for integrin inhibitors [6]. ATN-161 is a non-RGD-based pentapeptide binding to *α*5*β*1- and *α*V*β*3-integrins, derived from fibronectin by replacing an arginine residue of the primary sequence with cysteine moiety [6]. It has been shown to inhibit tumor growth, angiogenesis, and metastasis in multiple animal models [28, 33]. In Phase I safety trials, ATN161 was well tolerated, and several patients exhibited stable disease, including one ovarian carcinoma [34]. Since the 1990s, *α*v*β*3-integrin has been identified as a target for antiangiogenic therapy, as it expresses in proliferating vascular endothelial cells and regulates endothelial cell migration in sprouting vessels [24]. LM609, a mouse anti-human monoclonal antibody raised against *α*V*β*3-integrin, showed considerable antiangiogenic activity in preclinical models [35]. As a result of these studies, etaracizumab (MEDI-522), a humanized version of LM609, was developed as one of the first integrin antagonists introduced into clinical trials. However, clinical trials found it to have limited effectiveness as a metastatic cancer treatment, probably owing to the single integrin (*α*V*β*3-) targeting [6, 36]. The human *α*v-integrin specific monoclonal antibody, intetumumab (CNTO-95), which targets both *α*v*β*3- and *α*v*β*5-integrins, also showed antitumor and antiangiogenic effects in xenograft tumor models [37, 38]. In a Phase I clinical trial, intetumumab was nontoxic, localized to tumors, and showed signs of antitumor activity [39]. A complete response imaged by FDG-PET was observed in one patient with ovarian carcinosarcoma whose disease remained stable for 6 months while receiving intetumumab [40]. This antibody should be further evaluated in additional clinical trials. Among various available RGD mimic peptides, cilengitide (c-[RGDf(NMe)V-]) has emerged as a promising agent. It can bind to both *α*V*β*3- and *α*V*β*5-integrins with high affinity and inhibit their function strongly [6]. Cilengitide has shown significant promise in patients with late-stage glioblastoma by extending patient survival with minimal side effects [41, 42]. It is currently being tested in Phase II trials in patients with lung and prostate cancer, and Phase II and Phase III trials are currently underway for glioblastoma [3]. However, in moving toward ovarian cancer clinical trials with cilengitide, a serious concern needs to be addressed. As noted above, Kaur et al. suggested that increased *α*v*β*3-integrin expression on ovarian cancer cells correlates with a favorable outcome and that inhibiting its activity could increase the severity of the disease [26]. Therefore, it is critical to further investigate and clarify the effects of anti-*α*v*β*3-integrin therapy on ovarian cancer tumors and the surrounding endothelial cells, before embarking on clinical therapeutic trials [18].

## 6. Conclusion

Recognition of the need for cytoreduction along with the evolution of surgical techniques and the establishment of chemotherapy regimens through multiple clinical trials allows a majority of ovarian cancer patients to achieve “disease-free” status after the initial treatment. One of the major disappointments with the current ovarian cancer treatments is failure to achieve a complete cure, even in optimally debulked or chemosensitive patients. The establishment of efficacious consolidation or maintenance therapies would be a powerful tool for improving the miserable outcomes of patients with advanced-stage disease.

The biological behavior of ovarian carcinoma is unique, differing from the classic and well-studied pattern of hematogenous metastasis found in most other cancers. Once ovarian cancer cells have detached as single cells or clusters from the primary ovarian tumor, they are carried by the physiological movement of peritoneal fluid and finally metastasize to the peritoneum and omentum, suggesting that the attachment of cancer cells onto the mesothelial cells covering the basement membrane is the initial key step in metastasis. Bevacizumab has already shown significant utility in ovarian cancer treatment not only in combination with current chemotherapy but also as a single agent, indicating that antiangiogenic therapy has considerable promise. Given that targeting integrins can affect not only the diverse functions of tumor cells, including adhesion, migration, invasion, proliferation, and survival, but also tumor microenvironments, especially the angiogenic endothelial cells, integrin inhibitors obviously have the potential for clinical use in the near future. Unfortunately, although several clinical trials have been attempted against ovarian cancer, no integrin inhibitor has shown sufficiently promising efficacy to progress to further clinical investigation; the agents targeting only a single integrin, such as *α*v*β*3 and *α*5*β*1, failed to show evident clinical benefits in metastatic cancer treatment. In cancer progression, more than one integrin pathway is involved. For example, even if inhibition of the function of *α*5*β*1-integrin as a fibronectin receptor could be adequately achieved, the other integrins, such as *α*v*β*3 or *α*3*β*1, would eventually compensate for its function. Therefore, a combination of different integrin receptor pathways is likely to be more effective in the clinical setting and should be explored for the future clinical application.

Collectively, although there remain many questions and challenges, integrin-targeted therapies continue to be a promising approach to improve the outcomes of women with ovarian cancer.

## Figures and Tables

**Table 1:** Candidate integrin inhibitors for ovarian cancer treatment.

Drug name	Type	Target	Preclinical data in gynecologic cancer	Manufacturer	Ref.
Volociximab (M200)	Chimeric antibody	*α*5*β*1	*i.p*. treatment reduced tumor burden and ascites in SKOV-3ip1 ovarian cancer mouse xenografts by 83% and 97%, respectively.	Protein Design Labs	[20]

ATN-161	Peptide	*α*5*β*1	*i.v.* (1 mg/kg) injection inhibited the outgrowth of metastases at lung, liver, or spleen in a metastasis model mouse of MDA-MB-231 breast cancer cell lines.	Attenuon LLC	[28]

Etaracizumab (MEDI-522)	Humanized antibody	*α*v*β*3	*i.p*. treatment decreased tumor burden in the SKOV3ip1 and the HeyA8 mouse models by 36 and 49%, respectively and reduced the number of proliferating cells but not microvessel density.	Medimmune	[29]

Intetumumab (CNTO95)	Human antibody	*α*v*β*3 *α*v*β*5	Low doses (0.15–1.25 *μ*g/mL) of intetumumab were effective in inhibiting adhesion and migration of 6 uterine serous papillary carcinoma cell lines i*n vitro. *	Centocor	[30]

Cilengitide (EMD-121974)	Peptide	*α*v*β*3 *α*v*β*5	*α*v*β*3-integrin overexpression on SKOV3ip1 cells impaired invasion, protease expression, and colony formation *in vitro*. Cilengitide may have detrimental effects against ovarian cancer.	Merck KGaA	[26]
